# P-1430. Use of Patient-Informed Resources Tailored to Diverse Populations Living with HIV to Improve Shared Decision-Making (SDM)

**DOI:** 10.1093/ofid/ofae631.1605

**Published:** 2025-01-29

**Authors:** Ryan W Nall, Michelle Collins-Ogle, William R Short, Sandra Ann Springer, Leah Molloy, Laura Simone, Chris Napolitan, Jeffrey D Carter, Jenniffer A Meza Jimenez, Kelly E Pillinger

**Affiliations:** University of Florida, Gainesville, Florida; Children's Hospital at Montefiore / Einstein College of Medicine, Bronx, New York; University of Pennsylvania, Philadelphia, Pennsylvania; Yale University, New Haven, Connecticut; PRIME Education, Brighton, Michigan; PRIME Education, LLC, Fort Lauderdale, Florida; PRIME Education, Brighton, Michigan; PRIME Education, LLC, Fort Lauderdale, Florida; PRIME Education, Brighton, Michigan; PRIME Education, Brighton, Michigan

## Abstract

**Background:**

People with HIV (PWH) who are engaged in decision-making with their provider are more likely to report treatment satisfaction. Yet, tailored SDM tools for diverse populations of PWH are lacking.

**Figure:**
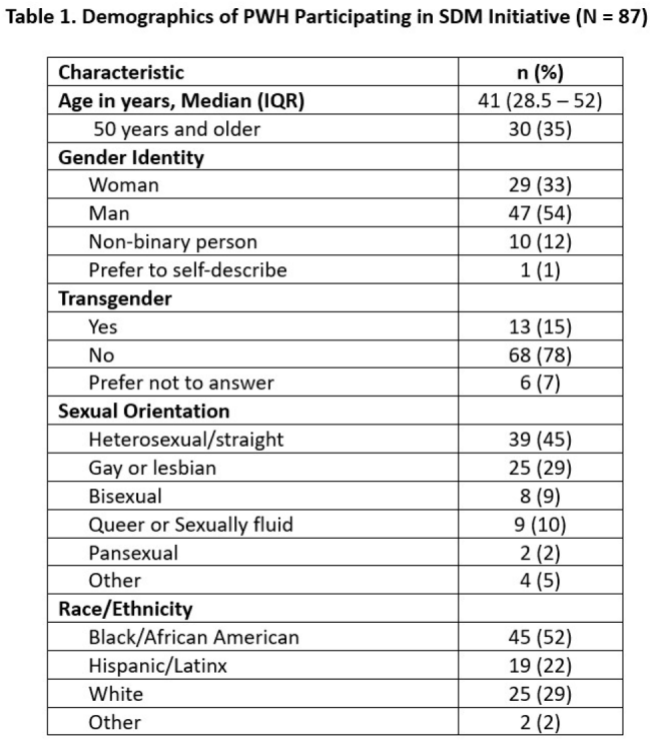

**Methods:**

Four steering committees of PWH and expert providers convened to develop SDM tools for diverse groups living with HIV, including gay and bisexual men, cisgender women, aging adults, and transgender individuals. From 9/23 – 12/23, the tools were implemented in 4 HIV clinics which entailed a short video introducing the concept of SDM and survey on a tablet during check-in, a paper conversation guide with prompts to identify specific health needs, and a survey at the end of the visit. An on-demand video describing SDM and the initiative’s results was disseminated to HCPs nationwide.

**Results:**

There were 87 PWH who participated in the use of the SDM tools (Table 1). A majority felt the SDM tools were helpful (83%) and were empowered to bring up questions about specific health care needs to their team (61% pre- vs. 74% post-survey; p = 0.063). 64% reported discussing their concerns and questions with their HIV care team more or much more than in previous visits, with 50% feeling very involved in decisions at their visit. Interestingly, a third of participants felt it was the care team’s role to make the decisions or they did not want to be involved in SDM. Of the 15 HCPs involved, 67% had previous formal training in SDM. They felt the biggest barriers to SDM were lack of time during visits (40%) and lack of patient interest (33%).

Of HCPs who participated in the on-demand video (N = 405), there was an increase in commitment to talking about barriers to staying engaged in HIV care (34% pre vs. 50% post; p < 0.001) and involving PWH in treatment decisions (45% pre vs. 62% post; p < 0.001). Top barriers to SDM among this group were low health literacy (38%), lack of professional training in SDM (28%), and scarcity of resources for PWH with diverse needs (27%). Confidence in engaging people from diverse backgrounds in SDM increased from 65% to 77% (p < 0.001).

**Conclusion:**

Implementation of SDM tools tailored to diverse populations of PWH are needed and can improve engagement in care. Reasons for lack of interest in SDM among some PWH should be explored further. Formal training in SDM for HCPs can increase confidence and use in real-world settings.

**Disclosures:**

**William R. Short, MD**, Gilead: Grant/Research Support|Janssen: Honoraria|ViiV Healthcare: Advisor/Consultant|ViiV Healthcare: Honoraria **Sandra Ann Springer, MD**, Alkermes Inc: Honoraria|Alkermes Inc: In kind study drug donation for NIH sponsored research|Indivior Pharmaceutical company: In kind study drug donation for NIH sponsored research **Kelly E. Pillinger, PharmD**, AHFS: Contractor

